# Potential Presymptomatic Transmission of SARS-CoV-2, Zhejiang Province, China, 2020

**DOI:** 10.3201/eid2605.200198

**Published:** 2020-05

**Authors:** Zhen-Dong Tong, An Tang, Ke-Feng Li, Peng Li, Hong-Ling Wang, Jing-Ping Yi, Yong-Li Zhang, Jian-Bo Yan

**Affiliations:** Zhoushan Center for Disease Control and Prevention, Zhoushan, China

**Keywords:** cluster, asymptomatic, coronavirus, 2019 novel coronavirus disease, COVID-19, severe acute respiratory syndrome coronavirus 2, SARS-CoV-2, viruses, respiratory infections, Zhejiang, China

## Abstract

We report a 2-family cluster of persons infected with severe acute respiratory syndrome coronavirus 2 in the city of Zhoushan, Zhejiang Province, China, during January 2020. The infections resulted from contact with an infected but potentially presymptomatic traveler from the city of Wuhan in Hubei Province.

In January 2020, we investigated a 2-family cluster of persons infected with severe acute respiratory syndrome coronavirus 2 (SARS-CoV-2) in the city of Zhoushan in Zhejiang Province, China. We attributed the infections to contact with an infected but potentially presymptomatic traveler from the city of Wuhan in Hubei Province. Our epidemiologic investigation was reviewed and approved by the Ethics Committee of the Zhoushan Centers for Disease Control and Prevention (CDC).

The initial 2 cases of SARS-CoV-2 infection (coronavirus disease [COVID-19]) in Zhoushan were diagnosed in 2 teachers (persons A and D) from the same department at a college that had sponsored an academic conference on January 5, 2020. A 45-year-old teacher from Wuhan (person W) arrived on January 5 for the conference and joined persons A and D on January 6 for dinner, where they ate from common serving plates. After returning to Wuhan on January 7, person W experienced the onset of fever, cough, sore throat, and malaise on January 8. He visited a local hospital where, according to the patient’s self-report, he was confirmed to have COVID-19 by a local office of the Chinese CDC. For person A and D, the only known potential exposures for SARS-CoV-2 were their dinner and conference attendance with person W (Figure).

On January 10, person A (a 29-year-old man) experienced the onset of fever, cough, and skin tingling and went to a local hospital for treatment. Laboratory tests at the hospital indicated leukopenia, and a real-time reverse transcription PCR (rRT-PCR) test for influenza A and B viruses was negative. The patient was given an antipyretic and some traditional medicines commonly used in China. After 3 days, his fever subsided, but his cough persisted. On January 15, the patient went to a different hospital, where routine blood test results were unremarkable but a chest radiograph revealed bilateral invasive lesions. He was prescribed amoxicillin and levofloxacin for 3 days. Because his cough did not improve, he was hospitalized for further evaluation. When the treating physician learned that the patient had had contact with a visitor from Wuhan before symptom onset, a throat swab specimen was sent for rRT-PCR testing for SARS-CoV-2 (*1*). On January 19, SARS-CoV-2 infection was confirmed at the laboratory of the Zhoushan CDC.

Person A lived with his 28-year-old wife (person B) and his 21-year-old sister (person C). The 2 women were confined at home for 14 days starting on the day of person A’s hospital admission. Because of their 10 days of contact with person A after his fever onset, their respiratory specimens were collected on January 20 by Zhoushan CDC staff for SARS-CoV-2 testing. Person B was confirmed on January 20 to be positive for SARS-CoV-2 infection by rRT-PCR but had no symptoms. Person C remained negative for SARS-CoV-2 during her quarantine period.

On January 12, person D (a 42-year-old man) experienced onset of low-grade fever, cough, and myalgia. He did not seek medical attention, and he traveled on business to the city of Sanya in Hainan Province during January 16–18. In Sanya, he used over-the-counter medications for his dry cough, which did not improve. On January 19, after returning to Zhoushan, he was informed that his colleague (person A) had been diagnosed with laboratory-confirmed COVID-19; person D was then transported by ambulance to the local infectious diseases hospital for isolation and specimen testing. That night, his throat swab specimen was confirmed to be weakly positive for SARS-CoV-2 by rRT-PCR.

Person D’s 41-year-old wife (person E) and his 12-year-old son (person F) were confined at home starting the day of person D’s hospital admission (January 19). On January 21, Zhoushan CDC staff collected respiratory specimens from persons E and F to test for SARS-CoV-2 infection, which was later confirmed by rRT-PCR, despite their lack of symptoms. On January 20, person W was interviewed by telephone by epidemiologists at Zhoushan CDC; he recalled having no prodromal or other symptoms on January 6, when he dined with persons A and D.

Our findings are subject to several limitations. The investigators conducted a telephone interview with the index patient (person W), who might have not recalled the full extent of his symptoms 14 days prior. Similarly, asymptomatic persons might have failed to report mild or subclinical symptoms. We cannot rule out other SARS-CoV-2 exposures to persons A and D in addition to their dinner and conference attendance with person W. Furthermore, we did not conduct convalescent serologic testing to provide additional evidence of the asymptomatic SARS-CoV-2 infections.

In summary, we identified 2 persons with confirmed cases of symptomatic COVID-19 after their exposure to a potentially presymptomatic person who was later diagnosed with laboratory-confirmed COVID-19. These 2 persons later transmitted SARS-CoV-2 to 3 family members, who did not report symptoms at the time their SARS-CoV-2 infections were detected.

**Figure:**
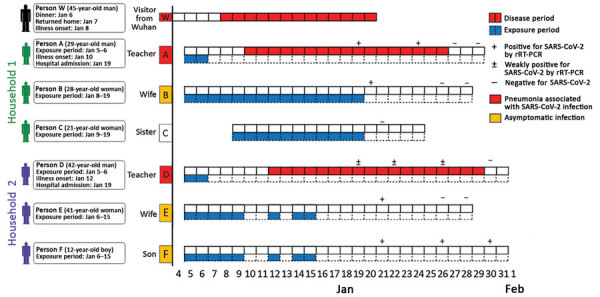
Epidemiologic linkage and timeline of severe acute respiratory syndrome coronavirus 2 infection within 2 households in the city of Zhoushan, Zhejiang Province, China, January 2020, and relationship to a visitor from Wuhan, Hubei Province, China (person W). Persons A and D had dinner with person W on January 6. During January 10–11, person D traveled on business to Guangzhou, Guangdong Province; on January 13, he traveled on business to Shanghai, Shanghai Municipality; and during January 16–18, he traveled on business to Sanya, Hainan Province. Therefore, gaps exist in exposure dates for person D. rRT-PCR, real-time reverse transcription PCR; SARS-CoV-2, severe acute respiratory syndrome coronavirus 2.
